# Physical Activity Reduces the Risk of Developing Diabetes and Diabetes Medication Use

**DOI:** 10.3390/healthcare10122479

**Published:** 2022-12-08

**Authors:** Ángel Denche-Zamorano, David Manuel Mendoza-Muñoz, Sabina Barrios-Fernandez, Carolina Perez-Corraliza, Juan Manuel Franco-García, Jorge Carlos-Vivas, Raquel Pastor-Cisneros, María Mendoza-Muñoz

**Affiliations:** 1 Promoting a Healthy Society Research Group (PHeSO), Faculty of Sport Sciences, University of Extremadura, 10003 Caceres, Spain; 2 Occupation, Participation, Sustainability and Quality of Life (Ability Research Group), Nursing and Occupational Therapy College, University of Extremadura, 10003 Caceres, Spain; 3 Research Group on Physical and Health Literacy and Health-Related Quality of Life, Centro Universitario de Plasencia, Universidad de Extremadura, 10600 Plasencia, Spain; 4 Health, Economy, Motricity and Education (HEME) Research Group, Faculty of Sport Sciences, University of Extremadura, 10003 Caceres, Spain; 5 Physical Activity for Education, Performance and Health, Faculty of Sport Sciences, University of Extremadura, 10003 Caceres, Spain; 6 Research Group on Physical and Health Literacy and Health-Related Quality of Life (PHYQOL), Faculty of Sport Sciences, University of Extremadura, 10003 Caceres, Spain; 7 Departamento de Desporto e Saúde, Escola de Saúde e Desenvolvimento Humano, Universidade de Évora, 7004-516 Évora, Portugal

**Keywords:** Diabetes, medication, physical activity, physical heath

## Abstract

Diabetes is a global public health challenge, exerting a large socioeconomic burden on healthcare systems. This study aimed to explore Diabetes prevalence and Diabetes medication use in diabetics regarding sex, age group, Physical Activity Level (PAL) and Body Mass Index (BMI) by studying possible differences and calculating the risks of developing Diabetes and Diabetes medication use in the population according to their PAL. A cross-sectional study was conducted using data extracted from the Spanish National Health Survey (ENSE2017). The sample was finally composed of 17,710 participants. A descriptive analysis was performed to characterise Diabetes prevalence and Diabetes medication use (Chi-square test and a z-test for independent proportions). Odds Ratios (OR) and 95% Confidence Intervals (CI) were calculated for Diabetes prevalence and Diabetes medication use according to the participants’ PAL. Both the Diabetes and Diabetes medication use was higher in men than in women, increasing with age and BMI, and decreasing with increasing PAL (*p* < 0.001). Higher prevalence levels were observed in the inactive group versus very active or active people (*p* < 0.001). Inactive people had a higher risk of Diabetes and use of Diabetes medication risk compared to the very active and active groups. Prevalence decreased the higher the PAL both in men and women.

## 1. Introduction

Diabetes mellitus is a chronic disease which has emerged as a significant public health challenge worldwide, both in developed and emerging countries [1]. In 2015, 415 million people were estimated to be living with Diabetes (8.8% worldwide). The has data doubled in the instance of those with Diabetes since 2000 (4.6%, 151 million people), which is an estimated increase to 10.4% (642 million) by 2040 [2]. Furthermore, in 2019, this disease was the ninth leading cause of death, responsible for 1.5 million deaths, 48% in people under 70. Between 2000 and 2016, premature mortality due to Diabetes increased by 5% [3]. As a result, Diabetes was considered a major socio-economic burden for many countries [4]. In Spain, the total annual direct cost of Diabetes amounts to EUR 5809 million, 8.2% of the total healthcare expenditure; pharmacological costs exerted the greatest influence on the total direct cost (38%) with EUR 2232 million per year [5]. Moreover, Diabetes has become a major cause of cardiovascular disease, non-traumatic lower limb amputations, blindness, kidney failure and death worldwide [6]. Furthermore, an association between Diabetes and cancer has been demonstrated, with Diabetes considered a risk factor for cancer in all locations, with a stronger impact on men than women [7]. 

There is a significant number of people with Diabetes undiagnosed. In Europe, 37.9% of individuals belong to this group, which could mean that around 22 million individuals with an increased risk of developing cardiovascular disease are unaware of their condition [8]. In Spain, it was found that almost half of the cases detected were undiagnosed [9]. Although there are several types of Diabetes, Diabetes mellitus types 1 (DM1) and 2 (DM2) are the most common [10]. DM1 is associated with deficits in insulin production and requires daily administration of insulin [11], while DM2 results from a decrease in insulin production due to insulin resistance. Symptoms are similar in both types of Diabetes (thirst, excessive excretion of urine, constant hunger, visual disturbances, etc.), but are less intense in DM2 [12]. Between 5–10% of cases have DM1, while the remaining 90–95% have DM2 which can be controllable and/or improved by physical activity and healthy lifestyle promotion [13].

Non-pharmacological interventions such as physical activity (PA) performance and a healthy diet are considered promising methods in the prevention and control of this disease, reducing the socio-economic cost associated with its treatment [10]. Exercise improves blood glucose control in DM2, influences weight loss, reduces cardiovascular risk factors and improves well-being [14,15]. Performing regular exercise can prevent or delay the development of the disease [16] and, in the case of DM1, lead to improvements in insulin sensitivity, muscle strength and cardiovascular fitness [17]. In terms of types of exercise training, moderate/high-volume aerobic is associated with lower cardiovascular and mortality risks in both types of Diabetes [18]. Thus, regular PA increases cardiorespiratory fitness, reduces insulin resistance, and improves lipid levels, and endothelial function [19], while DM2 decreases A1C, blood pressure, insulin resistance and serum triglycerides [20]. Resistance exercise reports positive effects in reducing the exercise-induced hypoglycemia risk in DM1 [21], producing improvements in glycemic control, insulin resistance, strength, and BMI, and reducing blood pressure [22]. Nevertheless, current evidence shows that PA and diet adherence in diabetic patients is still lower than adherence to medication [16].

Therefore, this research aims (1) to explore Diabetes prevalence and Diabetes medication use related to sex, age group, Physical Activity Level (PAL) and Body Mass Index (BMI); (2) to study potential differences in the proportions of Diabetes prevalence and Diabetes medication use by the Physical Activity Level (PAL) according to sex, age and Body Mass Index (BMI); and (3) to estimate the Diabetes and Diabetes medication use risk probability risks in the population according to their Physical Activity Level (PAL).

## 2. Materials and Methods

### 2.1. Recruitment and Data Source

A cross-sectional study was conducted using data from the Spanish National Health Survey (ENSE2017), based on Diabetes prevalence and Diabetes medication use according to the PAL and by socio-demographic characteristics such as sex, age and BMI. The ENSE is a survey conducted every five years by the Ministry of Health, Consumer Affairs and Social Welfare (MSCBS) and the Spanish National Statistics Institute (INE), which aims to identify health status, indicators and socioeconomic factors of the population residing in Spain [23]. Trained and accredited interviewers conducted the surveys between October 2016 and October 2017 and published the data in June 2018. The ENSE2017 was the last one conducted before the COVID-19 pandemic. Moreover, the ENSE2017 follows a three-stage random sampling system by strata among individuals aged 15 years and older residents in Spain. Before the first stage, Spanish municipalities were grouped by strata, based on population size. In this first stage, municipalities were randomly selected from the strata. In the second stage, a random selection of dwellings was made and, in the third stage, a random selection of one of the residents of the dwelling, among those aged 15 and over, was performed. Subsequently, the selected sample was informed and asked for volunteers, keeping confidentiality and anonymous treatment of data. Finally, 23,089 participants responded to the ENSE2017 adult questionnaire [24]. These data were previously submitted and published as public and non-confidential files on the website of the MSCBS: https://www.sanidad.gob.es/estadisticas/microdatos.do (accessed on 10 May 2022).

### 2.2. Participants

**Figure 1:**
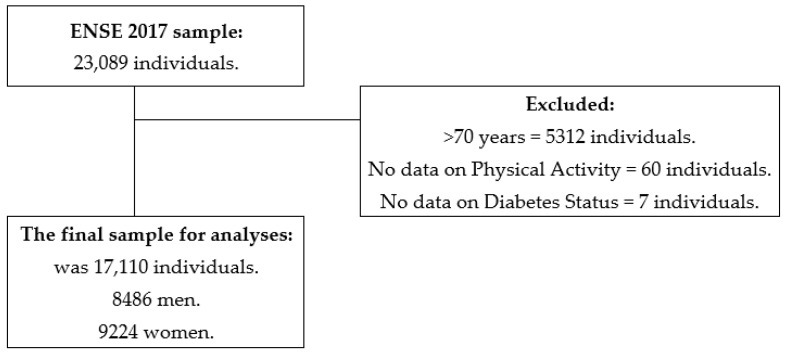
Chart outlining the study sample’s eligibility criteria.

The ENSE2017 complied with 23,089 participants’ data aged 15 years and older. Inclusion criteria were being younger than 70 years (as they were not questioned about their PA), providing all data on PA items (Q.113–Q.117) and Diabetes status (Q.25a.12). Thus, 5,312 participants were excluded for being older than 70 years, 60 for not having fulfilled items on PA and 7 for not completing Diabetes status data. Then, 17,710 participants’ responses were analysed, 8,486 men and 9,224 women. Figure 1 shows the flow chart with this process. For analyses that included Diabetes medication use, one participant who did not submit data was excluded (Q.87a.19). A total of 497 participants did not present data on the BMI Group variable; therefore, they were excluded in the analyses that included this variable. 

### 2.3. Variables

Sex: male or female.

Age: from which the variable “Age groups” was created with the following groups: 15–39, 35–49, 50–64 and 65–69 years.

BMI Groups: The ENSE2017 grouped participants according to their BMI into underweight (<18.5 kg/m^2^), normal weight (18.5 to 24.9 kg/m^2^), overweight (25 to 29.9 kg/m^2^), and obese (≥30 kg/m^2^).

Diabetes Status: extracted from the answers provided to item Q.25a.12 (“do you suffer, or have you ever suffered from Diabetes?”. Possible answers were “yes”, “no”, “I don’t know” or “no answer”.

Diabetes Medication Use: extracted from the answers to item Q.85 (“during the past two weeks, have you taken any medicines prescribed by a doctor?”. Possible answers were “yes” or “no”; and Q.87a.19 (“I will read a list of drugs, please tell me which one(s) you have taken in the last two weeks, and which ones have been prescribed by the doctor: Diabetes medicines?” Answers: “yes”, “no”, “I don’t know” or “not answer”. Participants were labelled as YES if they answered “yes” to items Q.85 and Q.85a.19, and NO if they answered “no” to item Q.85, “yes” to item Q.85 and “no” to item Q.87a.19.

Physical Activity Level (PAL): this variable grouped participants according to their PA level. For this purpose, the answers to items Q.113–Q.117 correspond to the International Physical Activity Questionnaire (IPAQ short form) in its Spanish version into a Physical Activity Index (PAI) [25]. The PAI could take values between 0 and 67.5, and this formula was described by Denche et al. [26] adapting the Nes et al. PAI, applying factors to the intensity (vigorous: 10; moderate: 5; light: 0), frequency (0 days/week: 0; One day/week: 1; Two or three days/week: 2; More than 3 days/week: 3) and duration (Less than 30 min: 1; 30 or more minutes: 1.5) that participants performed physical activity and calculating the sum [27]. Participants were grouped into four levels: Inactive (PAI = 0 and Q.117 = 0); Walkers (PAI = 0 and Q.117 > 0); Active (PAI between 1 and 30) and Very Active (PAI > 30); these groupings followed the indications of previous research [28].

### 2.4. Statistical analysis

The variables distribution was tested with the Kolmogorov–Smirnov test. The sample was characterised according to their Diabetes status and Diabetes medication use according to the general population, by sex, BMI, age and PAL group, reporting data in absolute and relative frequencies. Possible dependency relationships between Diabetes and medication use and socio-demographic variables were analysed with a Chi-square test. The relationship intensity was assessed using the contingency coefficient, interpreted according to Schubert [29]. Differences between Diabetes and prevalence of Diabetes medication use by the PAL were analysed with a pairwise z-test for independent proportions. Odds Ratios (OR) and their 95% confidence intervals (CI) for the Diabetes status and Diabetes medication use according to the PAL were calculated, taking the inactive group as a reference. Two multiple binary logistic regressions were performed taking as dependent variables the Diabetes status and the Diabetes medication use and sex, age, BMI and PAL as independent variables, analysing the predictor effects of these variables. The statistical software IBM SPSS Statistics for Windows, Version 25.0 (IBM Corp., Armonk, NY, USA) was used, considering two-sided *p*-values ≤ 0.05 as statistically significant.

## 3. Results

Table 1 shows the associations between Diabetes prevalence and sex, age group, PAL, and BMI (*p* < 0.001). There were more diabetic men than women (6.8% vs. 4.7%, *p* < 0.05). The Diabetes prevalence increased with age, being 0.6% in those under 34, and 18.3% in the 65–69 age group, with differences between all group proportions (*p* < 0.05). According to the PAL, the highest prevalence was found in the inactive and walker groups (8.0% and 7.4%) with no significant differences between them, decreasing to 2.2% in the very active group, *p* < 0.05. Diabetes was found in 0.5% of underweight people compared to 6.6% in overweight and 13.6% with obesity, with differences in the proportions in these groups (*p* < 0.05) and between them (*p* < 0.05).

Same dependency relationships were found between Diabetes medication use and sex, age, PAL and BMI (*p* < 0.001). Again, men showed higher Diabetes medication use than women (6.2% vs. 3.9%, *p* < 0.05). The under-35-year-olds had the lowest prevalence among the age groups (0.4%), with the 65–69-year-old group having the highest (16.3%), *p* < 0.05. Similarly, the inactive and very active groups had the highest and lowest prevalence according to PAL (7.2% vs. 1.7%, *p* < 0.05), while people with obesity (12.3%) had the highest among the BMI groups (Table 2). 

**Figure 2:**
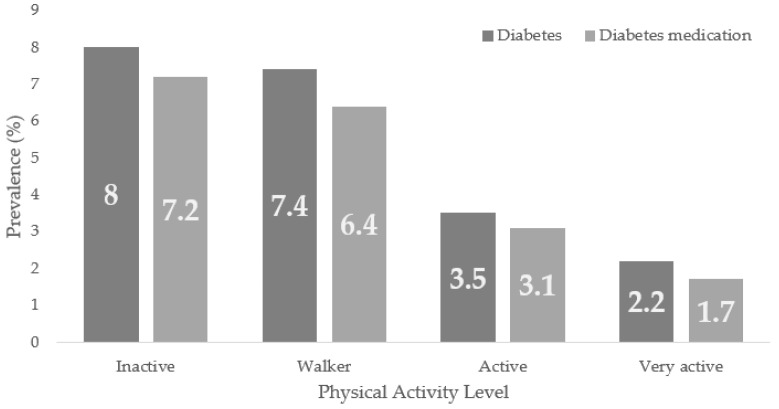
Diabetes and Diabetes Medication use Prevalence According to the Physical Activity Level (PAL) in the ENSE2017.

Figure 2 shows Diabetes and the prevalence of Diabetes medication use according to the PAL in the general population, being, in both cases, higher in the inactive group, with trends to lower prevalence as the population presents higher PAL.

Table 3 displays the Diabetes prevalence related to PAL in men and women from 50–64 years normal and overweight (*p* < 0.001). In all cases, the highest prevalence was found in the Inactive group. A decreasing trend in the Diabetes prevalence could be discerned as higher PAL was in sex, age, and BMI groups, but with no significant differences between the PALs. In men, the highest prevalence were found in inactive and walker (9.6%) groups with no differences between them. In contrast, the active (4.3%) and very active (2.3%) groups showed differences in proportions regarding the previous groups and with each other, *p* < 0.05. In females, the inactive and walker groups (6.6% and 5.8%) also had the highest Diabetes prevalence, with no significant differences between them, while there were significant differences between the active and very actives (2.6% and 2.2%), *p* < 0.05.

**Figure 3:**
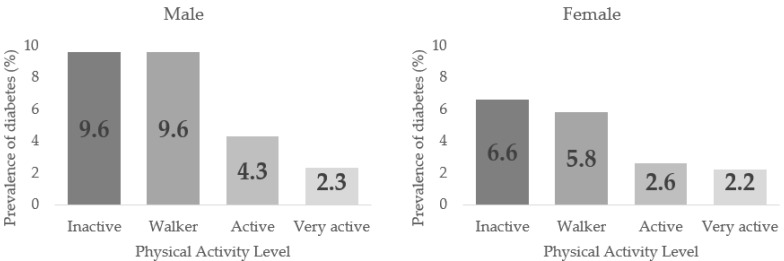
Diabetes Prevalence by Sex according to their Physical Activity Level (PAL).

Figure 3 shows the Diabetes prevalence in males and females according to the PAL.

Table 4 shows the associations between PAL and Diabetes prevalence by sex, age, and BMI groups. Significant dependence relationships were found between these variables in men, women, adults between 35–49, and in normal and overweight, *p* < 0.001. Prevalence were higher the lower the PAL, although no differences were found between the groups. Significant differences were found in women between the inactive/walker groups (5.5% and 4.8%, respectively) and the active/very active groups (2.1% and 1.4%) prevalence, *p* < 0.05. The same significant differences were found in men between the inactive/walker (9.0% and 8.6%), the active (4.1%) and the very active (1.8%) groups (*p* < 0.05), with significant differences also found between the latter two groups.

**Figure 4:**
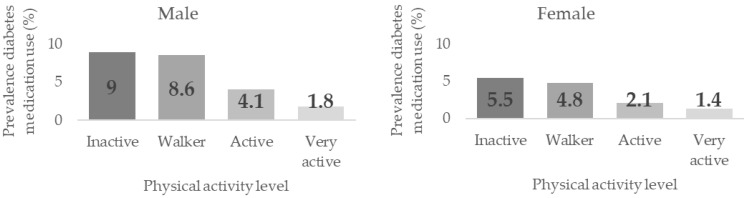
Diabetes Medication Use prevalence according to their Physical Activity Level by sex.

Figure 4 shows the Diabetes medication use prevalence according to the Physical Activity Level (PAL) in men and women.

Table 5 shows the Diabetes risk according to the PAL based on the inactive group. Significantly reduced risks were found in the active and very active groups compared to the inactive group in the general population, in both sexes, in the 35–49 and 50–64 years groups, and most BMI groups.

The Diabetes medication use risk was similarly reduced in the active and very active groups compared to the inactive ones (Table 6).

According to the binary multiple regression analysis on the Diabetes status and Diabetes medication use, older, male, inactive and obese people showed increased Diabetes and Diabetes medication use risks. These models explained 20.5% and 21.4% of the variance (Nagelkerke R^2^) in the Diabetes status and Diabetes medication use, respectively (Table 7).

## 4. Discussion

This study found associations between Diabetes prevalence and sex, with men having a higher prevalence than women, whose differences in proportions were statistically significant. One study carried out in the general population was in the same line, with a higher Diabetes prevalence in men than in women and increased prevalence in older people [30]. Similar results were found in the north-western European population, where the Diabetes mean prevalence was 5.9% for women and 7.9% for men [31]. In the study carried out by Aregbesola et al., a 61% higher risk in men rather than in women was found [32]. This higher prevalence in men could be justified by a higher accumulation of body iron in men and a limited capacity for subcutaneous fat expansion in men [33], so males would accumulate more fat in visceral (liver, spleen, and pancreas) and skeletal muscles, generating greater oxidation of the accumulated fats, leading to increased insulin resistance and glucose homeostasis disturbance [34,35]. Dependent relationships were also found between Diabetes prevalence and age groups. Diabetes prevalence increased with increasing age, with the highest prevalence (18.3%) in the older age group (65–69 years). The study performed by Bullard in the USA population, based on data from the 2016 National Health Interview Survey (NHIS) showed that DM2 prevalence increased progressively in the older age group; the highest prevalence was found among adults over 65 years and older [36]. 

Diabetes percentage was higher in the BMI groups, with the lowest percentage (0.5%) of diabetic patients in the underweight group and the highest percentage (13.6%) in the obesity group. These data were consistent with those of Zhao with the same group classification. However, percentages were established within the Diabetes group, with 0.54% in the low weight group and 61.94% in the obesity group [37]. The research conducted by Glovaci with adults with BMI above 40 kg/m^2^ showed a higher risk (OR = 7.37, 95 % CI: 6.39–8.5) of Diabetes diagnosis than those with a BMI within the normal range [38]. Obesity may be one of the most important predictors of DM2 as excess body fat and unfavourable body fat distribution lead to a state of chronic inflammation and insulin resistance, as well as impaired insulin secretion [39]. The most sedentary individuals had higher BMI, waist circumference, and increased systolic blood pressure was found [40]. Among the individuals with DM1, the active group had a lower BMI with a low obesity prevalence, a lower fat mass index and a lower waist circumference [41]. Our study obtained that Diabetes prevalence in obese subjects (BMI ≥ 30) was lower in the groups with higher PAL (Active 10.7 % and Very Active 6.5%) than in those with lower PAL (Inactive 15.2%), being these differences between proportions were significant. Therefore, there could be a strong association between sedentary behaviours (lower PAL) and higher BMI in diabetic and non-diabetic patients. Among the most sedentary individuals, generally, a higher BMI, waist circumference, and increased systolic blood pressure were found [42]. In individuals with DM1, the active group had lower BMI with a low obesity prevalence, lower fat mass index and a lower waist circumference [43]. The Diabetes prevalence in our study in Obese subjects (BMI ≥ 30) was lower in the groups with higher PAL (active 10.7% and very active 6.5%) than in those with lower PAL (inactive 15.2%), with these differences being significant proportions [42]. Therefore, there could be a strong association between sedentary behaviours (lower PAL) and higher BMI in both diabetic and non-diabetic individuals [42,43]. 

The highest Diabetes prevalence were found in the participants with the lowest PAL (inactive and walking groups) and the lowest in those with the highest PAL (active and very active groups). In this line, Colak used the IPAQ-SF Questionnaire [44] to assess PA and found that in 129 patients with DM2, 39.5% were Inactive and only 8.5% showed high PAL [45]. Oguntibeju found [46] reduced Diabetes risks in the active and very active groups compared to the inactive group in the general population, in both sexes, in the age groups 35–49 and 50–64 years, and most BMI groups. Other research has shown this inverse relationship between PA and Diabetes risk [47,48,49,50]. However, these studies used different instruments to assess PA (quantitatively, qualitatively, or mixed), measured different domains (occupational PA, leisure time PA, etc.) and dimensions (type of PA, intensity, duration, etc.), which could be problematic in making comparisons [51,52].

Diabetes medication use prevalence were higher in subjects with lower PAL, with prevalence decreasing the higher the PAL. In this regard, another study showed an association between a daily dose of vigorous exercise and lower odds of Diabetes medication use [53]. These findings were in line with those of the current study: the higher the PAL, the lower the medication prevalence. Therefore, diabetic individuals taking more medication have more severe symptoms of the disease and, consequently, experience greater challenges or barriers to physical activity or even prevent physical activity, possibly related to the adverse effects of the medication itself. 

The importance of this study lies in the analysis of the associations between the physical activity level and Diabetes prevalence and Diabetes medication use in the Spanish population during the last period before the COVID-19 pandemic, serving as a frame of reference for future research examining post-pandemic periods, as the ENSE is addressed every 5 years. This research showed the relationship between PAL and Diabetes. Hence, introducing exercise programs in Diabetes could be considered as a potential tool for its benefits on patients’ health such as improvements in glucose metabolism and insulin sensitivity [54], though this should be confirmed by studies that allow cause-effect relationships to be established.

This study has some limitations. On the one hand, a cross-sectional design does not allow for establishing cause–effect relations. Thus, further research using designs which allow causal relations to be established would be advisable. This work was based on self-reported Diabetes without any medical history or medical judgement. Additionally, this study lacked data on the type of Diabetes, which could influence the results. In future research, it would be interesting to use means of collecting objective data for Diabetes diagnosis. Moreover, additional sociocultural, socio-demographic, and socio-economic variables that could influence the results of Diabetes prevalence were also not considered [55,56,57].

## 5. Conclusions

Diabetes and Diabetes medication prevalence use was higher in men than in women and increased with age groups, with the highest prevalence levels among those aged 65–69 years and the lowest prevalence levels among those under the age of 34 years. These prevalence increased with the higher the BMI, with underweight people having the lowest prevalence levels and obese people the highest. The prevalence in men and women were highest in the inactive and walking groups and lowest in the active and very active groups, i.e., prevalence levels decreased the higher the PAL. Therefore, Diabetes risk and Diabetes medication use could be reduced the more active and very active a person is compared to inactive people in the general population in both sexes, in age groups of 35–49 and 50–64, and in most BMI groups.

## Figures and Tables

**Table 1:** Population Characteristics by their Diabetes Prevalence in the ENSE2017.

Characteristic	Overall	Diabetes		No Diabetes		X^2^	df	*p*	CC
	n	n	%	n	%				
Overall	17,710	1016	(5.7)	16,694	(94.3)	n.a	n.a.	n.a	n.a
Sex									
Men	8486	578 a	(6.8)	7908 a	(93.2)	34.8	1	<0.001	0.044
Women	9224	438 b	(4.7)	8786 b	(95.3)				
Age (years)									
15–34	3872	25 a	(0.6)	3847 a	(99.4)	950.6	3	<0.001	0.226
35–49	6176	137 b	(2.2)	6039 b	(97.8)				
50–64	5953	541 c	(9.1)	5412 c	(90.9)				
65–69	1709	313 d	(18.3)	1396 d	(81.7)				
PAL Group									
Inactive	2531	203 a	(8.0)	2328 a	(92.0)	160.3	3	<0.001	0.095
Walkers	8063	593 a	(7.4)	7470 a	(92.6)				
Actives	4888	171 b	(3.5)	4717 b	(96.5)				
Very actives	2228	49 c	(2.2)	2179 c	(97.8)				
BMI (kg/m^2^)	n = 17,213	n = 998		n = 16,225					
	n	n	%	n	%				
<18.5	415	2a	(0.5)	413	(99.5)	501.9	3	<0.001	0.168
[18.5–25)	7765	193a	(2.5)	7572 a	(97.5)				
[25–30)	6192	408b	(6.6)	5784 b	(93.4)				
>=30	2841	385c	(13.6)	2456 c	(86.4)				

Data presented in absolute and relative values; n: Participants; %: Percentage; PAL: Physical Activity Level; BMI: Body Mass Index; X^2^: Pearson’s Chi-square; df: degrees of freedom; *p*: *p*-value; CC: Contingency Coefficient; abcd: Different letters indicate significant differences between people with Diabetes proportions according to Sex, Age, BMI and Physical Activity Level groups; with *p* < 0.05 from pairwise z-test for independent proportions; n.a. not applicable.

**Table 2:** Population Characteristics by Diabetes Medication Use in ENSE2017.

Characteristic	Overall	Medication		No Medication		X^2^	df	*p*	CC
	n	n	%	n	%				
Overall	17709	886	(5.0)	16823	(95.0)	n.a	n.a.	n.a	n.a
Sex									
Men	8485	524 a	(6.2)	7961	(93.8)	47.1	1	<0.001	0.052
Women	9224	362 b	(3.9)	8862	(96.1)				
Age (years)									
15–34	3872	17 a	(0.4)	3855	(99.6)	879.5	3	<0.001	0.218
35–49	6176	110 b	(1.8)	6066	(98.2)				
50–64	5952	481 c	(8.1)	5741	(91.9)				
65–69	1709	278 d	(16.3)	1431	(83.7)				
PAL Group									
Inactive	2531	181 a	(7.2)	2350	(92.8)	146.9	3	<0.001	0.091
Walkers	8062	516 a	(6.4)	7546	(93.6)				
Actives	4888	152 b	(3.1)	4736	(96.9)				
Very actives	2228	37 c	(1.7)	2191	(98.3)				
BMI (kg/m^2^)	n = 17,212	n = 860		n = 16,352					
	n	n	%	n	%				
<18.5	415	2 a	(0.5)	413	(99.5)	493.2	3	<0.001	0.167
[18.5–25)	7765	153 a	(2.0)	7612	(98.0)				
[25–30)	6192	356 b	(5.7)	5836	(94.3)				
>=30	2840	349 c	(12.3)	2491	(87.7)				

Data presented in absolute and relative values; n: Participants; %: Percentage; PAL: Physical Activity Level; BMI: Body Mass Index; X^2^: Pearson’s Chi-square; df: degrees of freedom; *p*: *p*-value; CC: Contingency Coefficient; abcd: Different letters indicate significant differences between People with Diabetes proportions according to Sex, Age, BMI and Physical Activity Level groups; with *p* < 0.05 from pairwise z-test for independent proportions; n.a. not applicable.

**Table 3:** Diabetes prevalence through the Physical Activity Level (PAL) according to Sex, Age, and Body Mass Index (BMI).

	Physical Activity Level											
Variables	Inactive		Walkers		Active		Very Active					
Sex	n	%	n	%	n	%	n	%	X^2^	df	*p*	CC
Male	113 a	9.6	324 a	9.6	107 b	4.3	34 c	2.3	125.0	3	<0.001	0.120
Female	90 a	6.6	269 a	5.8	64 b	2.6	15 b	2.2	58.1	3	<0.001	0.079
Age (years)												
15–34	6 a	1.3	9 a	0.6	6 a	0.5	4 a	0.5	3.6	3	0.310	0.030
35–49	26 ab	2.8	73 b	2.8	29 a	1.6	9 a	1.1	14.5	3	0.002	0.048
50–64	115 a	13.1	318 a	10.2	84 b	5.8	24 b	4.7	52.5	3	<0.001	0.093
65–69	56 a	22.6	193 a	19.5	52 b	13.3	12 ab	15.0	11.0	3	0.012	0.080
BMI (kg/m^2^)												
<18.5	0 a	0.0	2 a	0.3	0 a	0.0	0 a	0.0	2.3	3	0.506	0.075
[18.5–25)	35 a	3.9	107 a	3.4	40 b	1.6	11 b	0.9	37.6	3	<0.001	0.069
[25–30)	67 a	7.9	242 a	8.3	74 b	4.4	25 b	3.3	42.0	3	<0.001	0.082
>=30	91 a	15.2	224 a	14.8	57 ab	10.7	13 b	6.5	15.7	3	0.001	0.074

n: participants; %: percentage; X^2^: Pearson’s Chi-square; df: degrees of freedom; *p*: *p*-value; CC: Contingency Coefficient; abc: different letters indicate significant differences between the Diabetes proportions according to their Physical Activity Level (PAL), *p* < 0.05 from pairwise z-test for independent proportions.

**Table 4:** Diabetes Medication Use prevalence by the Physical Activity Level (PAL) and Sex, Age and Body Mass Index (BMI).

	Physical Activity Level											
Variables	Inactive		Walkers		Active		Very Active					
Sex	n	%	n	%	n	%	n	%	X^2^	df	*p*	CC
Male	106 a	9.0	292a	8.6	100 b	4.1	26 c	1.8	119.3	3	<0.001	0.118
Female	75 a	5.5	224a	4.8	52 b	2.1	11 b	1.4	51.5	3	<0.001	0.075
Age (years)												
15–34	4 a	0.8	5a	0.4	5 a	0.4	3 a	0.4	2.1	3	0.553	0.023
35–49	23 ab	2.5	60b	2.3	22 ac	1.2	5 c	0.6	17.3	3	0.001	0.053
50–64	102 a	11.6	283a	9.1	78 b	5.4	18 b	3.5	47.4	3	<0.001	0.089
65–69	52 a	21.0	168ab	17.0	47 b	12.1	11 ab	13.8	9.8	3	0.020	0.076
BMI (kg/m^2^)												
<18.5	0 a	0.0	2a	1.0	0 a	0.0	0 a	0.0	2.3	3	0.506	0.075
[18.5–25)	30 a	3.3	82a	2.6	34 b	1.4	7 b	0.6	31.4	3	<0.001	0.063
[25–30)	57 a	6.7	216a	7.4	66 b	3.9	17 b	2.3	43.2	3	<0.001	0.084
>=30	84 a	14.1	201a	13.3	52 ab	9.8	12 b	6.0	13.7	3	0.003	0.069

n: participants; %: percentage; X^2^: Pearson’s Chi-square; df: degrees of freedom; *p*: *p*-value; CC: Contingency Coefficient; abc: different letters indicate significant differences between the Diabetes proportions according to their Physical Activity Level (PAL), *p* < 0.05 from pairwise z-test for independent proportions.

**Table 5:** Diabetes Risk According to the Physical Activity Level.

Physical Activity Level										
	Inactive	Walkers			Active			Very Active		
Variables		OR	CI95%		OR	CI95%		OR	CI95%	
Overall	Ref.	0.91	0.77	1.07	0.42	0.34	0.51	0.26	0.19	0.35
Sex										
Male	Ref.	0.99	0.70	1.24	0.42	0.32	0.56	0.22	0.15	0.33
Female	Ref.	0.86	0.67	1.10	0.38	0.28	0.53	0.28	0.16	0.49
Age Group										
Young	Ref.	0.51	0.18	1.43	0.39	0.12	1.21	0.39	0.11	1.38
Young adults	Ref.	1.02	0.65	1.61	0.56	0.33	0.95	0.39	0.18	0.83
Older adults	Ref.	0.75	0.00	0.94	0.41	0.31	0.55	0.32	0.21	0.51
Older	Ref.	0.83	0.59	1.16	0.53	0.35	0.80	0.61	0.31	1.20
BMI										
<18.5	Ref.	n.a.	n.a.	n.a.	n.a.	n.a.	n.a.	n.a.	n.a.	n.a.
[18.5–25)	Ref.	0.87	0.59	1.28	0.40	0.26	0.64	0.23	0.12	0.46
[25–30)	Ref.	1.06	0.80	1.40	0.54	0.38	0.76	0.40	0.25	0.64
>=30	Ref.	0.97	0.74	1.26	0.67	0.47	0.95	0.38	0.21	0.70

Ref: reference; OR: Odds Ratio, >1 higher risk of reporting Diabetes; CI95%: 95% OR Confidence Interval; n.a. not applicable.

**Table 6:** Diabetes Medication Use Risk according to the Physical Activity Level.

Physical Activity Levels										
	Inactive	Walkers			Active			Very Active		
Variables		OR	CI95%		OR	CI95%		OR	CI95%	
Overall	Ref.	0.89	0.74	1.06	0.42	0.33	0.52	0.22	0.15	0.31
Sex										
Male	Ref.	0.95	0.75	1.20	0.43	0.32	0.57	0.18	0.12	0.28
Female	Ref.	0.86	0.66	1.13	0.37	0.26	0.54	0.25	0.13	0.47
Age Group										
Young	Ref.	0.42	0.11	1.59	0.49	0.13	1.82	0.44	0.10	1.96
Young adults	Ref.	0.95	0.58	1.55	0.48	0.26	0.86	0.24	0.09	0.64
Older adults	Ref.	0.76	0.60	0.96	0.43	0.32	0.59	0.27	0.16	0.46
Older	Ref.	0.77	0.54	1.09	0.52	0.34	0.80	0.60	0.30	1.22
BMI										
<18.5	Ref.	n.a.	n.a.	n.a.	n.a.	n.a.	n.a.	n.a.	n.a.	n.a.
[18.5–25)	Ref.	0.77	0.51	1.18	0.40	0.25	0.66	0.17	0.07	0.39
[25–30)	Ref.	1.11	0.82	1.50	0.57	0.40	0.82	0.32	0.19	0.56
>=30	Ref.	0.94	0.71	1.23	0.66	0.46	0.96	0.39	0.21	0.73

Ref: reference; OR: Odds Ratio, >1 higher risk of reporting Diabetes; CI95%: 95% OR Confidence Interval; n.a. not applicable.

**Table 7:** Logistic Binary Regression Model for Diabetes and Diabetes Medication Use Risk Factor.

Diabetes								
	B	SE	Wald	df	Sig	Exp(B)	95% CI for EXP(B)	
							Lower	Upper
Sex (Women)	−0.358	0.071	25.764	1	0.000	0.699	0.609	0.803
Age	0.085	0.004	537.721	1	0.000	1.089	1.081	1.097
Inactive			42.017	3	0.000			
Walker	−0.167	0.092	3.317	1	0.069	0.846	0.706	1.013
Active	−0.587	0.114	26.535	1	0.000	0.556	0.445	0.695
Very active	−0.773	0.169	20.802	1	0.000	0.462	0.331	0.644
Underweight			187.363	3	0.000			
Normal	1.247	0.717	3.020	1	0.082	3.479	0.853	14.192
Overweight	1.782	0.716	6.192	1	0.013	5.939	1.460	24.162
Obesity	2.487	0.716	12.059	1	0.001	12.025	2.954	48.943
Constant	−8.609	0.744	133.738	1	0.000	0.000		
Diabetes Medication Use								
	B	SE	Wald	df	Sig	Exp(B)	95% CI for EXP(B)	
							Lower	Upper
Sex (Women)	−0.467	0.076	38.116	1	0.000	0.627	0.540	0.727
Age	0.090	0.004	493.403	1	0.000	1.094	1.085	1.103
Inactive			39.358	3	0.000			
Walker	−0.184	0.097	3.582	1	0.058	0.832	0.687	1.007
Active	−0.560	0.121	21.563	1	0.000	0.571	0.451	0.723
Very active	−0.947	0.192	24.208	1	0.000	0.388	0.266	0.566
Underweight			185.194	3	0.000			
Normal	0.967	0.719	1.808	1	0.179	2.630	0.643	10.761
Overweight	1.564	0.717	4.762	1	0.029	4.780	1.173	19.483
Obesity	2.305	0.717	10.337	1	0.001	10.027	2.459	40.878
Constant	−8.755	0.751	135.743	1	0.000	0.000		

B: understandarized beta; SE: standard error of the regression; Wald: Wald Chi-Squared Test; Df: degrees of freedom; Sig: statistical significance; Exp: exponential regression; CI: Confidence Interval).

## Data Availability

Datasets are available through the corresponding author upon reasonable request.
